# Supplementary material for: Content-rich biological network constructed by mining PubMed abstracts
Source: BMC Bioinformatics. 2004 Oct 8;5:147. doi: 10.1186/1471-2105-5-147 (PMC528731; doi:10.1186/1471-2105-5-147)
Supplement: Additional File 2 — The original results of the above study (non-essential files are deleted to keep the file size under the limit set by BMC bioinformatics). [file 1471-2105-5-147-S2.bz2 › chilibotAdditionalFile2/dip40/752ID8393934E18137/html/RUVA_RUVB.html]

 


 **RUVA** and **RUVB** 
  
Found 87 abstracts in PubMed, retrieved 40.  
 

 What does Google say? 
 PDF only 
| .edu only 

---

**Interactive relationship** (e.g. stimulation, inhibition, etc)

**Stimulatory relationship**- E. coli  **RuvA**  greatly enhanced the ability of Tth  **RuvB**  to hydrolyze ATP in the presence of DNA and to promote branch migration of a synthetic Holliday junction at 37 degrees C.  Ref: 10485292 Mol Gen Genet, 1999
- The purified Tth  **RuvA**  protein stimulated Tth  **RuvB**  activities, such as hydrolysis of ATP and promotion of branch migration of the Holliday junction, in a manner similar to the  **RuvA**   **RuvB**  interactions observed in E. coli.  Ref: 11245216 Genes Genet Syst, 2000
- In addition, Tth  **RuvA**  stimulated the E. coli  **RuvB**  activities in vitro, which was well consistent with the results of in vivo hetero complementation experiments.  Ref: 11245216 Genes Genet Syst, 2000
- **RuvA**  targets the junction and facilitates loading of  **RuvB**  helicase and RuvC endonuclease to form complexes that catalyse junction branch migration RuvAB and resolution RuvABC.  Ref: 11080172 EMBO J, 2000
- **RuvA**  binds to the Holliday junction and enhances the ATPase activity of  **RuvB**  required for branch migration.  Ref: 10772859 J Mol Biol, 2000
**Neutral relationship**- Functional interactions of Mycobacterium leprae  **RuvA**  with Escherichia coli  **RuvB**  and RuvC on holliday junctions.  Ref: 10966790 J Mol Biol, 2000
- this interaction is vital for the  **RuvA**  assisted loading of  **RuvB**  onto Holliday junction DNA.  Ref: 11427534 J Biol Chem, 2001
- In prokaryotes, the  **RuvA** , B, and C proteins play major roles at the late stage of DNA homologous recombination, where  **RuvB**  complexed with  **RuvA**  acts as an ATP dependent motor for branch migration.  Ref: 11042078 J Struct Biol, 2000
- Electron microscopic visualisation of the RuvAB complex revealed that  **RuvA**  is flanked by two  **RuvB**  hexamers, which bind DNA arms that lie diametrically opposed across the junction 8.  Ref: 10662672 Curr Biol, 2000
- **RuvA**  protein binds specifically to the Holliday junction and recruits  **RuvB**  protein to the junction.  Ref: 11245216 Genes Genet Syst, 2000
- A unique beta hairpin protruding from AAA ATPase domain of  **RuvB**  motor protein is involved in the interaction with  **RuvA**  DNA recognition protein for branch migration of Holliday junctions.  Ref: 11427534 J Biol Chem, 2001
- The Escherichia coli  **RuvB**  protein is a motor protein that forms a complex with  **RuvA**  and promotes branch migration of Holliday junctions during homologous recombination.  Ref: 11427534 J Biol Chem, 2001
- Individual roles for each protein have been defined, with  **RuvA**  acting as a specificity factor that targets  **RuvB** , the branch migration motor to the junction.  Ref: 9442895 Annu Rev Genet, 1997
- The sequence alignments of the Ruv proteins were extended by incorporation of data about the putative ruv genes obtained from data banks, and the  **RuvB**  sequences were conspicuously more conserved than the  **RuvA**  and RuvC sequences.  Ref: 8982068 Gene, 1996
- We previously cloned the  **ruvB**  gene of the thermophilic bacterium Thermus thermophilus HB8 Tth and found that, in contrast to the operon structure in most mesothermic bacteria, the  **ruvA**  gene is absent from the vicinity of  **ruvB** .  Ref: 11245216 Genes Genet Syst, 2000
- B. burgdorferi  **ruvB**  is part of an operon which comprises the homologues of  **ruvA** , queA and pfbB.  Ref: 9766225 Res Microbiol, 1998
- Specific binding of the  **RuvA**  tetramer to the Holliday junction is required for the  **RuvB**  motor protein to be loaded onto the junction DNA, and the RuvAB complex drives the ATP dependent branch migration.  Ref: 10890893 Proc Natl Acad Sci U S A, 2000
- These  **RuvB**  mutants hydrolyzed ATP and bound duplex DNA with the same efficiency as wild type  **RuvB** , but the mutants did not form a complex with  **RuvA**  and were defective in loading onto junction DNA in a  **RuvA**  assisted manner.  Ref: 11427534 J Biol Chem, 2001
- These results indicate the conservation of the  **RuvA**   **RuvB**  interaction in different bacterial species, and suggest the existence of a  **ruvA**  homolog in T. thermophilus.  Ref: 10485292 Mol Gen Genet, 1999
- Escherichia coli  **RuvB**  protein, an ATP dependent hexameric DNA helicase, acts together with  **RuvA**  protein to promote branch migration of Holliday junctions during homologous recombination and recombinational repair.  Ref: 10464259 J Biol Chem, 1999
- The presence of  **RuvB**  and the concentration of Mg2 both have a significant effect on cleavage in the presence of  **RuvA** .  Ref: 9000618 J Mol Biol, 1996
- In E.coli, branch migration is catalysed by the  **RuvB**  protein, a hexameric DNA helicase that is loaded onto the junction by  **RuvA** .  Ref: 9501105 EMBO J, 1998
- This process involves two key steps branch migration, catalysed by the  **RuvB**  protein that is targeted to the Holliday junction by the structure specific  **RuvA**  protein, and resolution, which is catalysed by the RuvC endonuclease.  Ref: 12423347 Eur J Biochem, 2002
- The atomic structure of  **RuvA**  should enable critical questions to be addressed about its specific interactions with the Holliday junction and the  **RuvB**  protein.  Ref: 9493263 Structure, 1998
- The E. coli  **RuvA**  and  **RuvB**  proteins, which are involved in the late stages of recombination and the recombinational repair of damaged DNA, bind to Holliday junctions and promote branch migration.  Ref: 9047358 J Mol Biol, 1997
- Two  **RuvA**  tetramers form the symmetric and closed octameric shell, where four  **RuvA**  domain IIIs spring out in the two opposite directions to be individually caught by a single  **RuvB** .  Ref: 12408833 Mol Cell, 2002
- The model of the  **RuvA**   **RuvB**  junction DNA ternary complex, constructed by fitting the X ray structure into the averaged electron microscopic images of the  **RuvA**   **RuvB**  junction, appears to be more compatible with the branch migration mode of a fixed  **RuvA**   **RuvB**  interaction than with a rotational interaction mode.  Ref: 12408833 Mol Cell, 2002
- **RuvA**  has a well defined DNA binding surface that is sculptured specifically to accommodate a Holliday junction and allow subsequent loading of  **RuvB**  and RuvC.  Ref: 11874468 Eur J Biochem, 2002
- The Rhizobium etli  **ruvA**  and  **ruvB**  genes were cloned through a PCR based approach, using degenerate primers matching conserved sectors in the amino acid sequences of  **RuvB**  from eight bacterial species.  Ref: 10675620 Gene, 2000
- Biochemical and structural studies of the RuvAB Holliday junction complex have shown that  **RuvA**  binds directly to the Holliday junction 2 3 4 5 6 and acts as a specificity factor that promotes the targeting of  **RuvB**  7 8, a hexameric ring protein that drives branch migration 9 10 11.  Ref: 10662672 Curr Biol, 2000
- Together with the detailed structural information, the biochemical assays of various mutant  **RuvA**  proteins and domains, isolated by partial proteolysis, allowed us to define the functional roles of these domains in Holliday junction binding and the  **RuvB**  interaction.  Ref: 9493263 Structure, 1998

**Non-interactive relationship** (e.g. studied together, co-existance, homology, etc.)

- In association with  **RuvA** , the E. coli  **RuvB**  protein a hexameric ring helicase, promotes the branch migration of Holliday junctions during genetic recombination and DNA repair.  Ref: 9299321 J Mol Biol, 1997
- In the major pathway of homologous DNA recombination in prokaryotic cells, the Holliday junction intermediate is processed through its association with  **RuvA** ,  **RuvB** , and RuvC proteins.  Ref: 10890893 Proc Natl Acad Sci U S A, 2000
- The  **RuvA**   **RuvB**  protein complex facilitates branch migration of the junction, depending on ATP hydrolysis.  Ref: 9493263 Structure, 1998
- The deduced amino acid sequences of P. aeruginosa  **RuvA** ,  **RuvB**  and RuvC proteins were 5 2 and 55% identical to those of the corresponding E. coli Ruv proteins.  Ref: 8982068 Gene, 1996
- The single amino acid substitution resulted in major alterations to the biochemical activities of  **RuvB** , such that RuvBD113N was defective in DNA binding and ATP hydrolysis, while retaining the ability to form hexameric rings and interact with  **RuvA** .  Ref: 9299321 J Mol Biol, 1997
- Branch migration of Holliday junctions, which are central DNA intermediates in homologous recombination, is promoted by the  **RuvA**   **RuvB**  protein complex, and the junctions are resolved by the action of the RuvC protein in Escherichia coli.  Ref: 10485292 Mol Gen Genet, 1999
- Monoclonal antibodies directed against  **RuvA** ,  **RuvB** , or RuvC inhibited resolution in the reconstituted system.  Ref: 9160752 Cell, 1997
- Since a  **RuvA**  protein has not yet been identified in T. thermophilus, we used E. coli  **RuvA**  to examine the effects of  **RuvA**  on the activities of Tth  **RuvB** .  Ref: 10485292 Mol Gen Genet, 1999
- Modulation of  **RuvB**  function by the mobile domain III of the Holliday junction recognition protein  **RuvA** .  Ref: 10772859 J Mol Biol, 2000
- BACKGROUND All the  **ruvA** ,  **ruvB**  and ruvC mutants of Escherichia coli are sensitive to treatments that damage DNA, and are mildly defective in homologous recombination.  Ref: 9663656 Genes Cells, 1998
- Crystal structure of the  **RuvA**   **RuvB**  complex a structural basis for the Holliday junction migrating motor machinery.  Ref: 12408833 Mol Cell, 2002
- Expression of the B. burgdorferi  **ruvB**  and  **ruvA**  genes renders a wild type Escherichia coli sensitive to UV light and mitomycin, indicative of negative complementation.  Ref: 9766225 Res Microbiol, 1998
- In P. aeruginosa, the ruv region seemed to form a non SOS regulated single operon consisting of orf26 ruvC  **ruvA**   **ruvB** , while in this region of E. coli,  **ruvA**  and  **ruvB**  form an SOS regulated operon, orf26 and ruvC form a non SOS operon, and these two operons are split by orf23.  Ref: 8982068 Gene, 1996
- RuvAB catalyses the unwinding of RNA DNA partial duplexes of at least 218 bp in a reaction that requires both  **RuvA**  and  **RuvB** , ATP and Mg2.  Ref: 8980680 J Mol Biol, 1996
- The Escherichia coli  **ruvA**  and  **ruvB**  genes constitute an SOS regulated operon.  Ref: 11245216 Genes Genet Syst, 2000
- Biochemical studies have shown that  **RuvA**  and  **RuvB**  promote branch migration whereas RuvC resolves junctions by endonucleolytic cleavage.  Ref: 9710535 J Mol Biol, 1998
- This study describes the characteristics of two  **RuvB**  mutants, I148T and I150T, that do not promote branch migration in the presence of  **RuvA** .  Ref: 11427534 J Biol Chem, 2001
- In Escherichia coli, a Holliday junction is processed into recombinant products by the concerted activities of the  **RuvA**  and  **RuvB**  proteins, which together drive branch migration, and RuvC endonuclease, which resolves the structure.  Ref: 9512524 Nucleic Acids Res, 1998
- In addition, Tth  **RuvB** , in the absence of  **RuvA**  protein, promoted branch migration of a synthetic Holliday junction at 60 degrees C in an ATP dependent manner.  Ref: 10485292 Mol Gen Genet, 1999
- Loss of lambda prophage recombinogenicity in UV irradiated Escherichia coli the role of host genes  **ruvA** ,  **ruvB** , ruvC, and recG.  Ref: 11766962 Res Microbiol, 2001
- In prokaryotes,  **RuvA**   **RuvB**  complexes play a crucial role in the migration of the Holliday junction, which is a key intermediate of homologous recombination.  Ref: 10772859 J Mol Biol, 2000
- However, we could not find an SOS promoter and ORF for  **RuvA**  protein in the sequence upstream of  **ruvB**  in contrast to the E. coli homologue.  Ref: 9447236 Mutat Res, 1997
- The Escherichia coli  **RuvA**  and  **RuvB**  proteins promote the branch migration of Holliday junctions during the late stages of homologous recombination and DNA repair reviewed in 1.  Ref: 10662672 Curr Biol, 2000
- They are processed in Escherichia coli by the  **RuvA** ,  **RuvB**  and RuvC proteins.  Ref: 11080172 EMBO J, 2000
- In Escherichia coli, the products of the  **ruvA** ,  **ruvB**  and ruvC genes are all involved in the processing of recombination intermediates Holliday structures into recombinant molecules.  Ref: 8982068 Gene, 1996
- Irradiated prophage retained its ability to recombine in  **ruvA** ,  **ruvB** , ruvC, and recG mutants.  Ref: 11766962 Res Microbiol, 2001
- This modulation may facilitate the dynamic coupling between  **RuvA**  and  **RuvB**  during branch migration.  Ref: 10772859 J Mol Biol, 2000
- **RuvA**  and  **RuvB**  form a complex that interacts with Holliday junctions crossed DNA structures that are recombination intermediates and promotes branch migration.  Ref: 9637927 Curr Biol, 1998
- In Escherichia coli cells, the  **RuvA** ,  **RuvB**  and RuvC proteins participate in the processing of an important intermediate, the Holliday junction.  Ref: 9493263 Structure, 1998
- In Escherichia coli, the  **RuvA** ,  **RuvB**  and RuvC proteins are required for the late stages of homologous recombination and DNA repair.  Ref: 9637927 Curr Biol, 1998
- **RuvA**  and  **RuvB**  promote branch migration of Holliday junctions, a process that extends heteroduplex DNA.  Ref: 9973614 Nucleic Acids Res, 1999
- The  **RuvA** ,  **RuvB**  and RuvC proteins of Escherichia coli process Holliday junctions during genetic recombination and DNA repair.  Ref: 9710535 J Mol Biol, 1998
- The  **RuvA** ,  **RuvB** , and RuvC proteins in Escherichia coli play important roles in the late stages of homologous genetic recombination and the recombinational repair of damaged DNA.  Ref: 9442895 Annu Rev Genet, 1997
- Replacements of yacF, yacG, yacH, yacK cueO, yacL,  **ruvA** ,  **ruvB** , yabB, and yabC made with the cassette were used to verify its properties.  Ref: 12142427 J Bacteriol, 2002
- Previous studies indicate that RuvAB increases the rate at which heteroduplex products are formed by RecA, that  **RuvA**  and  **RuvB**  are required for this stimulation,.  Ref: 11106642 J Biol Chem, 2001
- Comparative analysis of the predicted polypeptides for  **RuvA**  and  **RuvB**  of R. etli showed highly conserved blocks with the corresponding homologs in other bacteria.  Ref: 10675620 Gene, 2000
- In Escherichia coli, the  **RuvA**  and  **RuvB**  proteins interact at Holliday junctions to promote branch migration leading to the formation of heteroduplex DNA.  Ref: 10543946 J Mol Biol, 1999
- **RuvA**  provides junction binding specificity and  **RuvB**  drives ATP dependent branch migration.  Ref: 10543946 J Mol Biol, 1999
- Although the  **ruvA**  and  **ruvB**  genes are contiguous in R. etli, complementation studies suggest that they are expressed independently.  Ref: 10675620 Gene, 2000
- The previous mutational and proteolytic analyses suggested that mutations in a carboxyl terminal domain domain III impair binding of  **RuvA**  to  **RuvB** .  Ref: 10772859 J Mol Biol, 2000
- The requirement for recA, recB, recC,  **ruvA** ,  **ruvB** , ruvC, and possibly recG gene expression indicates that recombination is essential for viability of dam bacteria probably to repair DNA double strand breaks.  Ref: 10629194 J Bacteriol, 2000
- We have used electron microscopy and image analysis to examine  **RuvA**  and  **RuvB**  bound to model Holliday structures.  Ref: 9047358 J Mol Biol, 1997
- Double mutants of Escherichia coli dam DNA adenine methyltransferase strains with  **ruvA** ,  **ruvB** , or ruvC could not be constructed.  Ref: 10629194 J Bacteriol, 2000
- The  **RuvA** ,  **RuvB**  and RuvC proteins form a complex on Holliday junction DNA that drives coupled branch migration RuvAB and resolution RuvC reactions.  Ref: 11080453 J Mol Biol, 2000
- The  **RuvA**  and  **RuvB**  proteins of Escherichia coli form an important part of this machinery.  Ref: 8918940 J Mol Biol, 1996
- Escherichia coli  **RuvB**  protein, together with  **RuvA** , promotes branch migration of Holliday junctions during homologous recombination and recombination repair.  Ref: 10844644 Mol Microbiol, 2000
- We present the X ray structure of the  **RuvA**   **RuvB**  complex, which plays a crucial role in ATP dependent branch migration.  Ref: 12408833 Mol Cell, 2002
- Two proteins,  **RuvA**  and  **RuvB** , form a complex that promotes ATP dependent branch migration of Holliday junctions, a process that is important for the formation of heteroduplex DNA.  Ref: 9442895 Annu Rev Genet, 1997
- In this work, we have analysed the ability of  **RuvA**  and  **RuvB**  to bypass large tracts of DNA heterology in both three and four strand exchange reactions, using recombination intermediates made by the E. coli RecA protein.  Ref: 8918940 J Mol Biol, 1996
- In this system, RecA protein formed recombination intermediates that were processed by the actions of the  **RuvA** ,  **RuvB** , and RuvC proteins.  Ref: 9160752 Cell, 1997
- In prokaryotes, branch migration and Holliday junction resolution are catalyzed by the  **RuvA**  and  **RuvB**  proteins, which form a complex with RuvC resolvase to form a resolvasome.  Ref: 11207366 Cell, 2001
- Molecular analysis of the Pseudomonas aeruginosa genes,  **ruvA** ,  **ruvB**  and ruvC, involved in processing of homologous recombination intermediates.  Ref: 8982068 Gene, 1996
- Using a synthetic Holliday junction, a multisubunit complex containing the junction and  **RuvA** ,  **RuvB**  and RuvC was detected.  Ref: 9637927 Curr Biol, 1998
- The  **RuvA** ,  **RuvB**  and RuvC proteins of Escherichia coli act together to process Holliday junctions formed during recombination and DNA repair.  Ref: 11874468 Eur J Biochem, 2002
- Whereas high concentrations of  **RuvB**  in the absence of  **RuvA**  can promote homologous branch migration, we find that  **RuvB**  alone is unable to catalyse heterologous bypass, indicating an essential role for both proteins in homologous recombination and recombinational DNA repair processes.  Ref: 8918940 J Mol Biol, 1996
- These results show that the three Ruv proteins work in a coordinated manner to promote Holliday junction resolution, and account for the resolvase defective phenotype exhibited by  **ruvA** ,  **ruvB**  or ruvC mutant strains.  Ref: 9710535 J Mol Biol, 1998
